# Basin-scale biogeography of *Prochlorococcus* and SAR11 ecotype replication

**DOI:** 10.1038/s41396-022-01332-6

**Published:** 2022-10-22

**Authors:** Alyse A. Larkin, George I. Hagstrom, Melissa L. Brock, Nathan S. Garcia, Adam C. Martiny

**Affiliations:** 1 Department of Earth System Science, University of California, Irvine, CA USA; 2 Department of Ecology and Evolutionary Biology, Princeton University, Princeton, NJ USA; 3 Department of Ecology and Evolutionary Biology, University of California, Irvine, CA USA

**Keywords:** Microbial ecology, Microbial biooceanography, Metagenomics

## Abstract

Establishing links between microbial diversity and environmental processes requires resolving the high degree of functional variation among closely related lineages or ecotypes. Here, we implement and validate an improved metagenomic approach that estimates the spatial biogeography and environmental regulation of ecotype-specific replication patterns (*R*_Obs_) across ocean regions. A total of 719 metagenomes were analyzed from meridional Bio-GO-SHIP sections in the Atlantic and Indian Ocean. Accounting for sequencing bias and anchoring replication estimates in genome structure were critical for identifying physiologically relevant biological signals. For example, ecotypes within the dominant marine cyanobacteria *Prochlorococcus* exhibited distinct diel cycles in *R*_Obs_ that peaked between 19:00–22:00. Additionally, both *Prochlorococcus* ecotypes and ecotypes within the highly abundant heterotroph *Pelagibacter* (SAR11) demonstrated systematic biogeographies in *R*_Obs_ that differed from spatial patterns in relative abundance. Finally, *R*_Obs_ was significantly regulated by nutrient stress and temperature, and explained by differences in the genomic potential for nutrient transport, energy production, cell wall structure, and replication. Our results suggest that our new approach to estimating replication is reflective of gross population growth. Moreover, this work reveals that the interaction between adaptation and environmental change drives systematic variability in replication patterns across ocean basins that is ecotype-specific, adding an activity-based dimension to our understanding of microbial niche space.

## Introduction

Over the past 20 years the field of microbial ecology has increasingly demonstrated that there is high genomic variability between closely related microbial taxa [1–3]. In the marine environment, this microbial diversity demonstrates systematic biogeographic patterns on a global scale [4–6]. However, linking diversity with taxa-specific functional roles remains a challenge. For example, the highly abundant cyanobacterium *Prochlorococcus* and the heterotroph *Pelagibacter* (SAR11) are composed of phylogenetic clades that are known to partition marine niche space at high levels of phylogenetic resolution and are, thus, often referred to as “ecotypes” [7–9]. *Prochlorococcus* ecotype distributions are well-aligned with temperature growth optima for cultured isolates [10]. However, very little is known about the in situ multidimensional regulation of ecotype-level activity and its relationship with the distribution of *Prochlorococcus* or SAR11. In fact, evidence suggests that the relationship between activity and distribution can be decoupled by trophic interactions [11, 12]. Inferences into metabolic activity may both provide an added dimension to microbial niche dynamics and reveal the linkages between genome content and taxa-specific functional roles.

Differences in the environmental control of activity among closely related taxa is poorly characterized. The environmental parameters that regulate the activity, growth, diversity, and distribution of microbial populations operate at differing timescales. In the marine environment, variability in light, temperature, and nutrient availability on the timescale of weeks to years structures microbial biogeography [13–15]. However, on shorter timescales, it cannot be assumed that the environmental drivers that influence microbial distribution equally impact growth. Specifically, constraints imposed by biotic and abiotic decay processes, such as grazing, physical mixing, and export, may result in a decoupling between growth and abundance [16]. As such, there is a need to characterize genotype-specific growth and activity in mixed populations to better understand the environmental factors structuring microbial ecosystem processes.

A variety of methods have been developed for linking metabolic activity with genotype variation in microorganisms. Metrics of relative cellular activity [17–20] exhibit strong relationships with microbial abundances [21–23]. In addition, techniques that use radioisotope labeling combined with imaging or sequencing can provide direct relationships between biogeochemical cycling and microbial genotypes [24–27], but are often laborious and are either limited to coarse phylogenetic resolution or small population size [28]. Novel computational methods estimating genotype-specific replication from metagenomics allow for large-scale comparisons. Bioinformatic approaches estimate prokaryotic replication by calculating relative gene copies at the origin versus the terminus of replication (i.e., the “peak-to-trough ratio” or PTR) [29–32]. While such approaches demonstrate strong correlations between replication and growth rates in vivo and have been applied to reduced diversity microbial systems (e.g., groundwater), more complex communities exhibit tenuous relationships between isolate growth and PTR [33] including in marine environments. The conflicting implications of metagenomic replication may be due to the limitation of ranking and reordering gene coverages to calculate PTR or the slope of coverage, which decouples replication estimates from genomic structure. Moreover, previous studies have either not accounted for diel replication patterns exhibited by light-synchronized microbial communities, or been unable to identify strong diel synchrony in cultured isolate strains [34]. In contrast, in situ communities of *Prochlorococcus* and *Synechococcus* show strong diel synchrony in replication and cell division based on DNA content and cell size [16, 35, 36]. Moreover, even studies that have struggled to relate PTR to net growth rates have suggested that it may be an appropriate indicator of relative activity in oligotrophic systems [33, 34]. We hypothesize that bioinformatic techniques using metagenomic coverage to estimate replication must account for time of sampling and sequencing bias, as well as be strictly linked to well-characterized genome structure in order to accurately reflect gross population growth patterns.

In this study, we evaluate how the replication of specific genotypes is controlled at large scales across wide biogeochemical gradients. Specifically, we capitalize on the well-characterized ecotypes of *Prochlorococcus* and SAR11 to link adaptation with activity at refined genetic scales. Our aims are to (i) improve and test a metagenomic estimate of microbial replication in marine environments and (ii) apply this method to determine the biogeography of ecotype-specific activity across ocean basins.

## Materials and methods

### Environmental parameters

Environmental measurements were collected via GO-SHIP standard operating procedures along the I09N transect from 22 March to 24 April 2016, which traversed from Perth, Australia (31° 02′ 01″ S, 110° 27′ 28″ E) to the Bay of Bengal (16° 44′ 15″ N, 90° 08′ 77″ E), and the I07N transect from 25 April to 4 June 2018, which traversed from Durban, South Africa (29° 52′ 04″ S, 31° 03′ 60″ E) to the Arabian Sea (17° 59′ 55″ N, 68° 00′ 22″ E). Underway temperature and salinity measurements were made with a mounted near-surface thermosalinograph. Whole water column inorganic nutrients (NO_3_^−^, NO_2_^−^, PO_4_^3−^, SiO_3_^2−^) were collected via a Niskin rosette system every latitudinal degree. At all Indian Ocean GO-SHIP stations, nutricline depth was calculated via linear interpolation as the depth at which [NO_3_^−^] was equal to 1.0 μM. At underway sampling locations, an average nutricline depth was interpolated from the GO-SHIP station before and after the sampling site. At stations outside of the GO-SHIP transect, including the first 15 collection sites for both I09N and I07N, World Ocean Atlas climatological nitrate depth profiles were used to calculate the nutricline depth. For GO-SHIP nutrient analysis protocols, I09N environmental data, and I07N environmental data, please see https://cchdo.ucsd.edu/.

To compare replication estimates to primary production and cellular abundance along the I09N transect, *Prochlorococcus* cell counts as measured by flow cytometry and primary production as measured by ^13^C-bicarbonate uptake were retrieved from [37]. Concurrent flow cytometry and C uptake measurements were collected from 22 surface stations. C uptake (nmol C L^−1^) was normalized by the proportion of daylight during the incubation (Cρ, nmol C L^−1^ percent daylight^−1^). Additionally, in order to estimate C uptake per cell and compare to *Prochlorococcus* cellular replication, Cρ was divided by *Prochlorococcus* cell counts (cells ml^−1^).

The Atlantic Ocean samples were originally planned for the GO-SHIP A13.5 transect, however, this transect was canceled enroute due to the onset COVID-19 pandemic. The resulting transect, referred to as “C13.5,” traversed from Cape Town, South Africa (34° 26′ 35.9″ S 17° 08′ 27.0″ E) to Norfolk, Virginia, USA (36° 05′ 27.4″ N 74° 34′ 04.2″ W) from March 21 to April 16, 2020. Underway temperature and salinity measurements were made with a mounted near-surface thermosalinograph. Niskin rosette deployments were canceled on this cruise, therefore, World Ocean Atlas seasonal average nitrate depth profiles were used to calculate the nutricline depth for the entire cruise. For limited A12/A13.5/C13.5 environmental data, please see https://cchdo.ucsd.edu/.

### DNA sampling

A total of 242 DNA samples were collected from the ship’s circulating seawater system every 4–6 h on I09N. Collection and extraction protocols for I09N DNA have been previously described in [38]. For I07N, a total of 197 4 L surface water samples were collected every 4 h from the ship’s circulating seawater system (intake depth ~7 m) for DNA analysis. An additional 51 DNA samples were collected from the Niskin rosette surface bottle (~5 m depth). Rosette sample volume ranged from 2.5–4 L due to variable water budgets. On C13.5 229 DNA samples (5–10 L) were collected from the ship’s circulating seawater system every 2–4 h between 07:00–22:00. All DNA samples were filtered through 0.22 μm pore size Sterivex filters (Millipore, Darmstadt, Germany) using sterilized tubing and a Masterflex peristaltic pump (Cole-Parmer, Vernon Hills, IL). DNA was preserved with 1620 μL of lysis buffer (23.4 mg mL^−1^ NaCl, 257 mg mL^−1^ sucrose, 50 mmol L^−1^ Tris-HCl, 20 mmol L^−1^ EDTA) and stored at −20 °C until analysis.

### Library preparation

DNA extraction and metagenomic library prep are described in [39] and in Supplemental Methods. Briefly, DNA was extracted via a lysozyme and Proteinase K incubation, precipitated, resuspended, purified using a Zymo genomic DNA Clean and Concentrator kit (Zymo Research Corp., Irvine, CA), and diluted to 2 ng/μl. Next, a modified Illumina Nextera DNA protocol and custom Nextera 8 bp unique dual index (UDI) barcodes were used to create metagenomic libraries using a Tagment DNA Enzyme and Buffer Kit (Illumina, San Diego, CA; cat. No. 20034197) [40–42]. I09N, I07N, and C13.5 samples were pooled separately and sequenced on NovaSeq lanes using 150 bp paired-end chemistry with 300 cycles (Illumina, San Diego, CA). A total of 864 Gbp with a median 20.5 million reads per sample (3–81 million) was generated for I09N, a total of 936 Gbp with a median of 21.7 million reads per sample (1.6–94 million) was generated for I07N, and a total of 896 Gbp with a median of 19.6 million reads per sample (2.7–142.7 million) was generated for C13.5.

### Metagenome analysis

Prior to analysis, Illumina adapters were removed and sequences were quality filtered using Trimmomatic (v0.35) and BBMap (v37.50). Next, Bowtie2 (v2.2.7) was used to map reads to a custom database of fully sequenced or >90% complete single amplified genomes of *Prochlorococcus* (39 genomes), *Synechococcus* (35 genomes), and SAR11 (34 genomes) that were evenly distributed throughout their respective phylogenetic trees. Additionally, a single genome from each of the SAR116, SAR406, SAR86, and *Roseobacter* clades were used as outgroups. Anvi’o (v5.5) was used to profile the recruited reads [43]. All open reading frames for all reference genomes were aligned and clustered using NCBI BLAST [44] and MCL [45] using the command anvi-pan-genome. Anvi’o was used to extract all single-copy core gene (SCCG) reads associated with the most abundant *Prochlorococcus* and SAR11 ecotypes using anvi-get-short-reads-mapping-to-a-gene and a minimum read length of 35 bp (–leeway 35) [43]. Finally, biopython (v1.76) and mySQL (v8.0) were used to convert SCCG reads into a database format. All non-default parameters for these steps are available in Supplemental Methods.

To account for differences in sequencing depth between samples, SCCG reads were rarefied to a single depth across all samples for each ecotype examined (Fig. S2). The rarefaction depth for each ecotype was selected based on reference genome length, such that the average genome coverage should be ≥5X, which has been shown to be the minimum depth necessary to calculate replication slope [29, 30]. The Python (v3.8.0) library pandas (v0.25.3) was used to randomly select ecotype-specific SCCG reads at each station using the dataframe.sample method. Next, the reference gene length, the summed read lengths, and the read count were used to calculate the SCCG coverage for each gene in each sample. The dataset was rarefied 30X before calculating the coverage slope.

### Replication estimate

To calculate ecotype-specific replication, the SCCG coverages for each ecotype were first ordered by a reference genome with high intra-ecotype synteny. We theorized that reference genomes with high synteny, i.e., conserved SCCG order compared to other genomes in the same ecotype, would represent an “average” gene order and be highly comparable to in situ populations. To select the reference genome with the fewest genome rearrangements, we calculated the double cut and join (DCJ) distance [46] for all reference genomes within an ecotype using UniMog software [47]. We then selected the top six genomes with the lowest mean DCJ. For SAR11 clade Ib, only two high-quality single cell genomes (>90% completion) were available as reference genomes at the time of the analysis; therefore, we also assessed the clade Ia genomes with the highest read recruitment as potential reference genomes for SCCG order-mapping. The top six genomes were clustered based on DCJ distance and the number of large gaps in SCCG mapping were counted (Fig. S1A). By assessing gaps in SCCG coverage, we attempted to minimize the number of genome rearrangements/hypervariable regions compared to in situ populations. Finally, the percentage of reads mapped to each reference genome was assessed as a proxy for genome similarity to in situ communities (Fig. S1B). Based on the (1) DCJ distance, (2) SCCG gap count, and (3) percent recruitment, a final reference genome was selected for each ecotype. Selected reference genomes included MED4 (*Pro*. HLI), MIT9301 (*Pro*. HLII), and HIMB083 (SAR11 1a). For SAR11 clade Ib, the two single cell reference genomes either had a high number of gaps (AG_337_G04) or no “V”-shaped coverage pattern (AG_430_F16). Therefore, clade Ia genome HIMB140 was selected as a gene order reference for clade Ib because it had low distance to AG_337_G04, shared 657 out of 817 SCCGs, and only demonstrated two gaps in SCCG coverage. A robust reference could not be identified for SAR11 clade II (Fig. S1) as mapping to HIMB058 genome resulted in a large number of gaps in SCCG coverage (Figure S2). Finally, for the *Prochlorococcus* HNLC clade (HLIII-IV), almost no samples reached the 5× coverage cutoff needed to calculate a replication slope (Fig. S2).

Next, the median circular minimum value of order-mapped SCCG coverage [29] was used to identify the putative terminus of replication across all samples. From the terminus, a non-linear least squares regression (“nls,” R “stats” package) was used to simultaneously fit linear models in the forward and reverse direction, such that the estimated slope of the linear fits was the same. The slope of coverage was calculated for each ecotype and across each station for 30X rarefactions (Fig. S2). Slopes were standardized by multiplying the linear slope by half the reference genome length. The mean standardized slope across all rarefactions was used in the following analyses, which we refer to as our ecotype-specific replication estimate, *R*_Obs_.

### Daily HLII maximum replication

To account for the diel pattern in replication for the *Prochlorococcus* HLII ecotype (Fig. S4), a model-based approach was developed to estimate daily maximum replication, or *R*_24hr,max._ (Fig. S5). We developed a hierarchical/multi-level model which predicts *R*_24hr,max._ using *R*_Obs_ and the measurement times, fitting unknown parameters using Bayesian statistics [48]. The model assumes the temporal variation of the expectation of *R*_Obs_ over the diel cycle follows a Gaussian function. Specifically, we model the value of each *R*_Obs_ as a normal distribution with mean given replication slope evaluated at the observation time, with a value of *R*_24hr,max._ distributed normally across each noon-to-noon bin, and with weakly informative priors on the replication time *t*_1_, the width of the replication period *t*_*w*_, the background replication slope *c*_0_, and the mean maximum replication slope *R*_24hr,max._ (see Supplemental Methods). The HLII *R*_24hr,max._ was similar to daily maximums estimated via linear interpolation (Fig. S6).

### Nutrient stress indicators

Macronutrients were below detection in a large proportion of the surface ocean. Thus, we used a genomic indicator (*Ω*) of adaptation to nutrient demand as a proxy for historical nutrient stress. These indicators are quantitatively related to surface nutrient concentrations and have been described in [49–51]. Briefly, genes were identified via the Anvi’o pangenomic workflow [52] through alignment, clustering, and annotation using NCBI BLAST and MCL. Gene clusters were then curated and target *Prochlorococcus* nutrient stress genes were selected. Nitrite and nitrate assimilation and uptake genes were designated as N-stress (*Ω*_N_), alkaline phosphatases were designated as P-stress (*Ω*_P_), and core HNLC genes (clade HLIII-IV) associated with the loss of Fe-containing proteins were designated as Fe-stress (*Ω*_Fe_). The coverage of each nutrient stress gene was normalized to summed *Prochlorococcus* SCCG coverage, transformed into a Z-score, and summed per nutrient stress category for comparison across transects. Increasing normalized coverage of nutrient stress indicators denotes an increased abundance of these genes in the environment and is indicative of phylogenomic adaptations to historically low nutrient availability.

### Machine learning analysis

We hypothesized that four environmental factors that would best explain replication patterns (*R*_Obs_ or *R*_24hr,max._) in SAR11 clades Ia and Ib and in *Prochlorococcus* HLII: temperature, nutricline depth (as a proxy for nutrient supply), historic P-stress (*Ω*_P_), and historic N-stress (*Ω*_N_). We examined both linear and non-linear effects of environmental factors on replication by implementing both linear (LM) and general additive models (GAM). Linear models were applied using the “boot.relimp” function in the “relaimpo” package, which assesses the relative importance of linear predictors by partitioning *R*^2^ over orders and assesses confidence via resampling of observations. To assess non-linear relationships, the “gam” function in the “mgcv” R package was used with thin plate regression spline smooths applied to each explanatory variable (temperature, nutricline depth, *Ω*_P_, *Ω*_N_) using the “s” function. To reduce overfitting and prevent arbitrary selection of the number of basis functions (k), the restricted maximum likelihood (“REML”) method was used to select smoothing parameters. The “gam.check” function was then used to assess whether the GAMs converged and whether residuals were randomly distributed. In the SAR11 Ia model, the residuals of the temperature parameter were non-random and therefore the *k* value was raised to 25. In all cases, the combined GAMs were significant and converged after 12–13 iterations. Finally, to assess the relative importance of predictors in each GAM, the reduction in deviance was calculated for each predictor via a symmetrized computation.

In order to identify genes that were predictive of *R*_Obs_ or *R*_24hr,max._we used a random-forest analysis of relative gene coverage. Only genes mapped to each ecotype that were not single-copy core genes were analyzed. Additionally, explanatory genes with no functional annotation tended to cluster near the origin of replication (data not shown), therefore were unlikely to have a functional relationship with replication, and were subsequently removed from the analysis. The “randomForest” function in the “randomForest” R package was used for this analysis. An initial fit was run with training dataset with 75% of the replication values randomly selected, 25 random explanatory variables (mtry = 25), assessment of predictors, and 2000 trees. We found that in all cases the mean square error of the model saturated at ~1000 trees, thus 2000 trees were sufficient for the identification of a robust model. Next, we optimized mtry by running the random-forest model across a range of mtry values and selecting the mtry with the lowest mean square error of the model. After the initial optimization of the number of trees and mtry, we ran the random-forest model 10 times to select all genes that were identified as the 40 most explanatory variables in the model by mean decrease in accuracy (MDA). With this first subset of genes we re-optimized mtry then performed back-selection of genes. Specifically, we iteratively ran the random-forest model fit, removed any genes with negative MDA values (i.e., non-significant for the model), re-optimized mtry, and re-ran the random forest until all genes had a positive MDA value. Finally, our test dataset (25% of replication values not selected for the training dataset) was run through the random-forest model to assess the predictive power of the model.

## Results and discussion

We first validated an optimized approach for estimating ecotype replication and then quantified the biogeography of replication across biogeochemical gradients. We collected 719 metagenomic libraries as part of Bio-GO-SHIP with samples from the Atlantic (C13.5) and Indian (I07N and I09N) sections from 36° N to 34° S. We quantified the metagenomic coverage of single-copy core genes (SCCGs) to determine both ecotype frequency and calculate the slope of gene coverage across reference genomes (i.e., metagenomically estimated replication). Next, we assessed diel and latitudinal trends in standardized replication (i.e., *R*_Obs_) and compared ecotype-level replication to environmental trends and functional gene content.

### Assessment of replication estimate

**Fig. 1:**
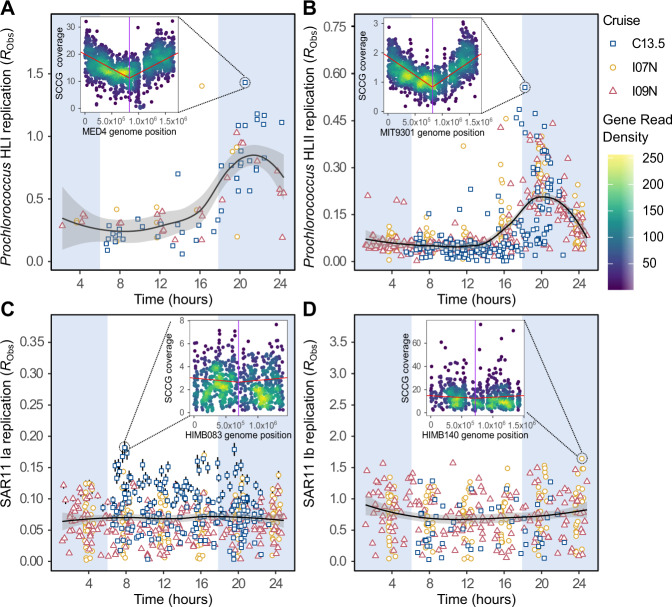
Ecotypes of the phototroph *Prochlorococcus* demonstrate distinct diel patterns in replication (*R*_Obs_), whereas ecotypes of the heterotroph SAR11 show consistent replication across the day-night cycle. Here, we examine the **A** HLI, **B** HLII, **C** clade Ia, and **D** clade Ib ecotypes. Point shape corresponds to C13.5 (squares), I07N (circles), and I09N (triangles) samples (with at least 5× coverage of single-copy core genes (SCCG) prior to rarefaction). Point whiskers represent the standard error of replication estimates across 30x rarefactions (standard error was low and is largely obscured by the points). The black trend line represents the central tendency of *R*_Obs_ patterns as characterized by a loess curve (“loess,” R “stats” package, span = 0.22) with 90% confidence intervals shaded in gray. White and blue background shading represents the light and dark diel cycle, respectively, with sunrise occurring at ~06:00 and sunset occurring at ~18:00. Insets: Single-copy core gene read coverage mapped to reference genome position for the station with the highest slope. The purple line is the terminus of replication. The red line is the bi-linear fit to the coverage pattern.

To account for varying genome structure, we standardized our replication estimate using reference genomes from the two most abundant marine bacteria—*Prochlorococcus* and *Pelagibacter*. Specifically, we identified the SCCG order of our reference genomes, used a single optimized reference genome with a fixed terminus for each ecotype, and assessed the role of variable sequence depth. The chosen reference genomes had high synteny (i.e., conserved SCCG order and low ‘double cut and join distance’) to other genomes within their respective ecotype, few gaps in SCCG coverage, and a high percentage of mapped reads (Fig. S1). This analysis suggested that the structures of these genomes were representative of both their broader ecotypes and in situ populations. When mapped to reference genomes with a fixed terminus of replication, SCCG coverage bias showed a bi-directional linear gradient for *Prochlorococcus* and SAR11 ecotypes (Figs. 1 and S4). Coverage of SCCGs was unlikely to be influenced by GC content sequencing bias, as average GC content was ~0.3 across ecotypes, only a small number of genes had GC content <0.2, and low GC content genes were evenly spread across reference genomes (Fig. S3). Another potential source of bias for calculating metagenomic replication is varying sequence depth. Indeed, sample read depth and coverage slope exhibited a significant relationship (*R*^2^ = 0.32–0.90) (Fig. S2). To account for this sequencing bias, we rarefied the SCCG reads associated with each ecotype to a single sequence depth across all stations. Although a fixed terminus resulted in negative slopes for a small proportion of samples (subsequently removed from analysis), we recommend the use of a single fixed terminus of replication for each ecotype across all samples in order to anchor metagenomic coverage in the cellular process of prokaryotic binary fission. Additionally, rarefaction reduced the likelihood that either sequencing bias or stochasticity resulted in an erroneous slope.

Estimated replication patterns in natural populations were consistent with a diel regulation of DNA replication. We estimated replication as genome length-standardized coverage slope averaged across all rarefactions (*R*_Obs_). Across natural *Prochlorococcus* HLII ecotype populations, *R*_Obs_ was low between 02:00–15:00, increased at ~16:00, and peaked at 20:00 (sunrise = ~06:00, sunset = ~18:00) (Fig. 1). This replication pattern matched previous observations wherein the maximum number of surface ocean *Prochlorococcus* cells in S-phase occurred at 20:00 [35, 53]. Despite lower overall genome coverage, ecotype HLI *R*_Obs_ also showed a diel pattern that peaked between 19:00–22:00. Thus, linking biases in metagenomic coverage with replication in *Prochlorococcus* required accounting for the daily cell cycle. We therefore introduced a daily peak replication slope (*R*_24hr,max._) estimated using a Bayesian-optimized tent function (Figs. S5 and S6). Autotrophic C uptake was significantly correlated to HLII *R*_24hr,max._ (*p* value < 0.05, Fig. S7), further linking this metric with cellular growth. Our data suggested that characterizing diel patterns is critical for placing metagenomically estimated coverage bias within the ecological context of daily *Prochlorococcus* replication.

In contrast to *Prochlorococcus*, we observed no significant diel rhythmicity in SAR11 Ia and Ib *R*_Obs_ (Fig. 1). To test this result, we also examined diel trends for SAR11 Ia across five sequence read depths (~5× to 15× average coverage) and found that increasing read depth did not influence our ability to detect a diel cycle (Fig. S8). The lack of a diel cycle in SAR11 likely contributed to the weaker bi-linear SCCG coverage bias, as sub-populations of cells were initiating replication throughout the day rather than during a synchronized time period. Nevertheless, bi-linear coverage bias for SAR11 (i.e., a “V”-shape with a single high-coverage origin and a single low-coverage terminus) was detected across all 24 h windows (Fig. S4). Overall, the bi-linear coverage bias patterns for both *Prochlorococcus* and SAR11 and the diel cycle of *R*_Obs_ in *Prochlorococcus* revealed a clear biological signal and a strong link between our replication estimate and the physiologically driven cycle of cellular division.

### Biogeography of ecotype replication

**Fig. 2:**
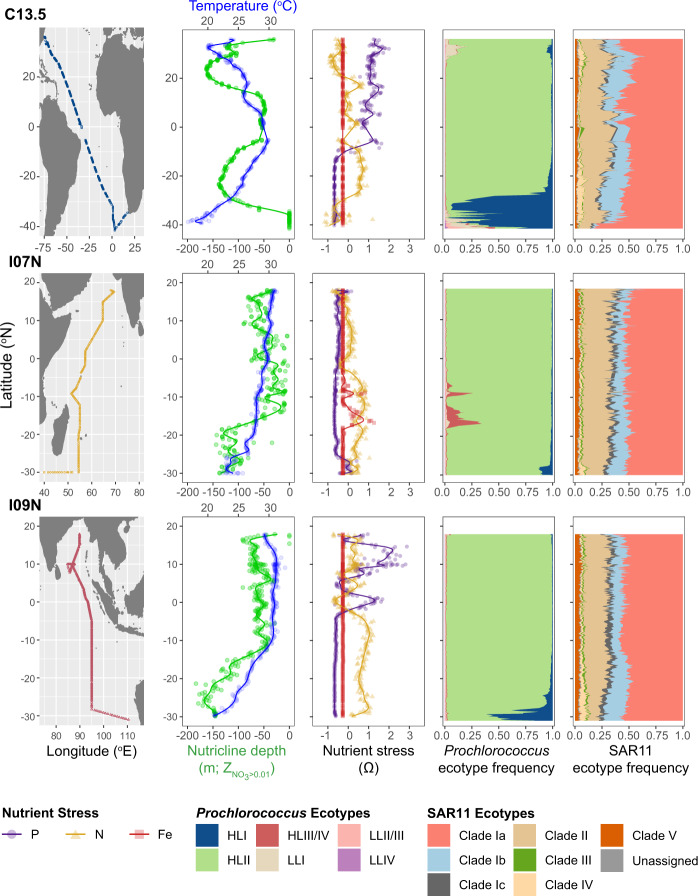
Basin-scale biogeography shows variable environmental patterns but stable ecotype frequencies. Measured environmental variables include temperature (blue) and interpolated nutricline depth (green) as well as N- (yellow), P- (purple), and Fe-stress (red) indices (*Ω*). Note that nutricline depth was measured via CTD cast aboard I07N and I09N but had to be estimated via World Ocean Atlas seasonal averages along the C13.5 transect due to COVID-19-driven cancellations. Frequency of *Prochlorococcus* and SAR11 ecotypes was measured by relative single-copy core gene (SCCG) coverage.

The frequency of *Prochlorococcus* and SAR11 ecotypes were largely stable across a wide range of environmental conditions. In the Atlantic, we observed a decoupling between temperature and nutricline depth across the spatial scale examined (Fig. 2). The Atlantic Ocean also had the widest diversity of the dominant type of genomically estimated nutrient stress (*Ω*) [51]. Across both Indian Ocean transects, temperature and nutricline depth were positively correlated and showed similar patterns, with temperatures above 25 °C across the majority of each transect and nutricline shoaling at ~15° S. However, the western basin had high Fe stress (*Ω*_Fe_) and the eastern basin showed a transition between high N and P stress (*Ω*_N_ and *Ω*_P_). Overall, each transect demonstrated unique environmental patterns.

Despite diverse combinations of nutritional and temperature environments, all three transects showed similar community ecotype structure. *Prochlorococcus* was dominated by the high light, high temperature ecotype HLII. Similar patterns were observed for the SAR11 clade Ia (~50% of SAR11) and clade II (~25% of SAR11) across a range of temperatures and nutrient stress conditions. SAR11 clade Ib frequency had the greatest variability and appeared to increase in the subtropical gyres of both basins. Additionally, unique communities were observed at the southern end of each transect (i.e, below 28° S), where the low-temperature ecotype HLI was observed. In addition, between ~5° S to 18° S along the I07N transect, the high-nutrient, low-chlorophyll ecotype HLIII/IV was observed concurrent with high Fe-stress. With a few exceptions, the frequency of each ecotype was stable over a wide gradient of temperature and nutrient stress regimes.

**Fig. 3:**
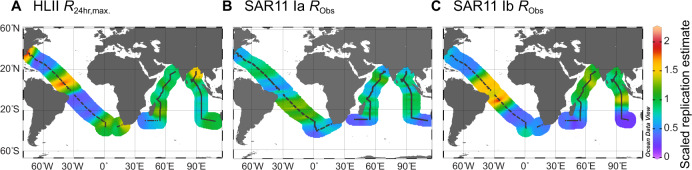
Large-scale biogeography of dominant *Prochlorococcus* and SAR11 lineages reveals ecotype-specific replication patterns. The black dots represent the sample locations and are missing where that clade fell below 5× coverage. *R*_Obs_ or *R*_24hr,max._ was centered and scaled for the **A** HLII, **B** clade Ia, and **C** clade Ib ecotypes on each cruise in order to compare relative replication patterns across ecotypes and sequencing runs.

**Fig. 4:**
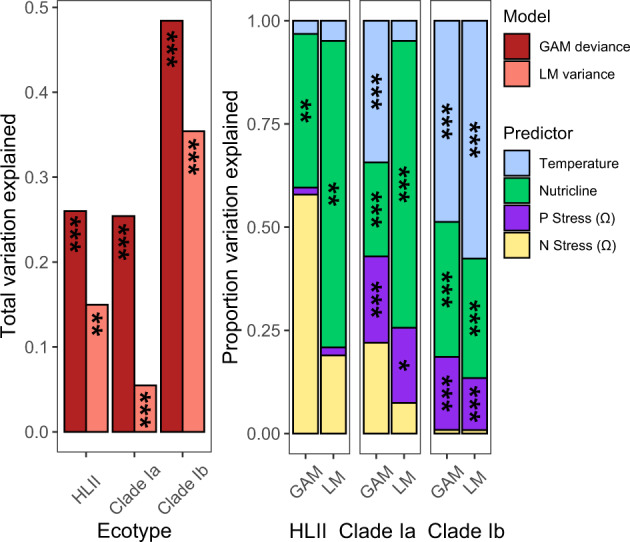
Linear (LM) and general additive models (GAM) reveal bottom-up controls of temperature and nutrient supply on replication. The combination of temperature (°C), nutricline depth (m, Z_NO3>0.01_), P stress (*Ω*_P_), and N stress (*Ω*_N_) explained significant variation in replication in both linear and non-linear models. The total variation explained by each model for each ecotype is depicted in the left panel for the GAM (red) and LM (pink) fits. For each model, the proportion of the total variation explained by each predictor (temperature – blue, nutricline depth – green, P stress – purple, N stress – yellow) as well as the significance of the predictor is depicted in the right panel (*p value < 0.05, **p value < 0.01, ***p value < 0.001).

Ecotype-specific replication demonstrated systematic biogeographic patterns and distinct regional partitioning. Examining replication at the ecotype level allowed us to parse population-specific patterns. When replication is assessed at the genus level, trends for *Prochlorococcus* are dominated by changes in ecotype frequency and trends for SAR11 demonstrate high stochasticity, suggesting that population-specific approach is needed to reveal systematic or underlying patterns (Figure S9). When assessed at the ecotype level, *Prochlorococcus* HLII replication was unique to each transect, likely reflecting local environmental conditions. In the Atlantic, HLII *R*_24hr,max._ was highest in the equatorial region, with additional peaks off the coasts of South Africa and North America, and was negatively correlated with nutricline depth (*r* = −0.35, *p* value < 0.001), suggesting a nutrient supply control (Figs. 2–4). This result is consistent with recent predictions of HLII growth patterns across the Atlantic [54]. In the western Indian Ocean basin, HLII *R*_24hr,max._ was consistently high, but decreased from 15°S to the equator, congruent with lower HLII frequency and elevated Fe-stress. In the eastern Indian Ocean, *R*_24hr,max._ for HLII was highest in the Bay of Bengal. The increase in *R*_24hr,max._ in the Bay of Bengal contrasted with stable HLII frequency (Fig. 2) and decreasing *Prochlorococcus* biomass in this region [37]. Due to the generally low genomic coverage of HLI, we were unable to apply our daily model to the HLI replication slopes. However, *R*_Obs_ between 19:00–22:00 was high near the Southern Subtropical Front with a steep drop-off at ~25–30°S where HLII replaced this ecotype (Fig. S9). SAR11 Ib showed biogeographic trends in *R*_Obs_ with some spatial similarities to HLII (*r* = 0.16, *p* value < 0.0001). For example, in the Atlantic Ocean, clade Ib had similar patterns to HLII, but peak *R*_Obs_ was shifted southwards coinciding with peak basin temperature. In the western Indian Ocean, SAR11 Ib *R*_Obs_ steadily increased from south to north, which was also consistent with temperature trends, but opposite to clade Ib frequency (Fig. 2). In the eastern Indian Ocean, clade Ib showed an increasing trend to ~10° S, before decreasing and demonstrating stable *R*_Obs_ in the northern hemisphere. SAR11 clade Ia had the most spatially stable replication patterns. Nevertheless, systematic biogeographic patterns in *R*_Obs_ were still observed. Specifically, SAR11 clade Ia showed elevated *R*_Obs_ in the subtropical gyres and repressed *R*_Obs_ in equatorial, high latitude, and coastal locations across all three transects (Figs. 3 and S9). Opposing estimated replication patterns between clade Ia and HLII in both the Atlantic and, to a lesser degree, the Indian Ocean resulted in a negative overall correlation (*r* = −0.10, *p* value < 0.05). In contrast, similarities in estimated replication patterns between clade Ia and clade Ib in the Indian Ocean drove an overall positive correlation (*r* = 0.23, *p* value < 0.0001). These patterns indicate that each ecotype may occupy a unique and spatially partitioned “activity niche” that is only partially related to ecotype frequencies.

Spatial variability in estimated ecotype replication was significantly explained by nutrient stress and temperature. General additive models explained more variation in replication than linear models, but the proportion of total variation explained by individual predictors was similar between non-linear and linear models (Fig. 4). We hypothesized that trends in *R*_24hr,max._ or *R*_Obs_ for HLII, SAR11 Ia, and SAR11 Ib would be best explained by a combination of elemental nutrient stress, nutricline depth, and temperature (Fig. 4). However, all three ecotypes did not have significant relationships between replication and *Ω*_N_. This result suggests that these ecotypes may be adapted to regions where nitrite and nitrate are chronically supplied at low rates [55]. In particular, HLII had highest replication at intermediate levels of *Ω*_N_, resulting in a high proportion of deviance explained in the non-linear GAM (Figs. 4 and  S10). All three ecotypes demonstrated negative relationships between replication and *Ω*_P_, though this relationship was non-significant for HLII. This may suggest that estimated SAR11 replication is constrained by the supply of organophosphate [56]. Although inorganic nitrogen and organic phosphorus stress did not have significant relationships with estimated HLII replication, nutricline depth did have a significant (*p* value < 0.01), negative, and linear (edf =1.00) relationship with *R*_24hr,max._ patterns, suggesting that nutrient supply plays an important role in HLII replication. In contrast, SAR11 Ia showed peak *R*_Obs_ at a nutricline depth of ~100–150 m and SAR11 Ib had peak *R*_Obs_ at nutricline depths of ~50–75 m (Figure S10). Both SAR11 clades showed increasing *R*_Obs_ with increasing temperature, with peak clade Ia *R*_Obs_ at 25–28 °C and peak clade Ib *R*_Obs_ at 28–30 °C. HLII *R*_24hr,max._ showed a non-significant negative relationship with temperature (Fig. 4). However, the observed temperature range of our daily HLII estimates was limited to 22–32 °C (Fig. S10). Finally, whereas the combined environmental factors explained 48.4% of deviance in estimated SAR11 Ib replication, only 26.0 and 25.4% of deviance in HLII and SAR11 Ia trends could be explained by the GAMs, respectively. Therefore, unmeasured and top-down controls [16] may play an important role in regulating the replication of these stable, dominant ecotypes. Overall, our analysis revealed that the ecotypes examined here had the highest estimated replication at intermediate-to-high temperature. Additionally, differing responses to nutrient supply appeared to be a key niche-defining parameter that regulated estimated replication patterns between both autotrophic and heterotrophic ecotypes.

**Fig. 5:**
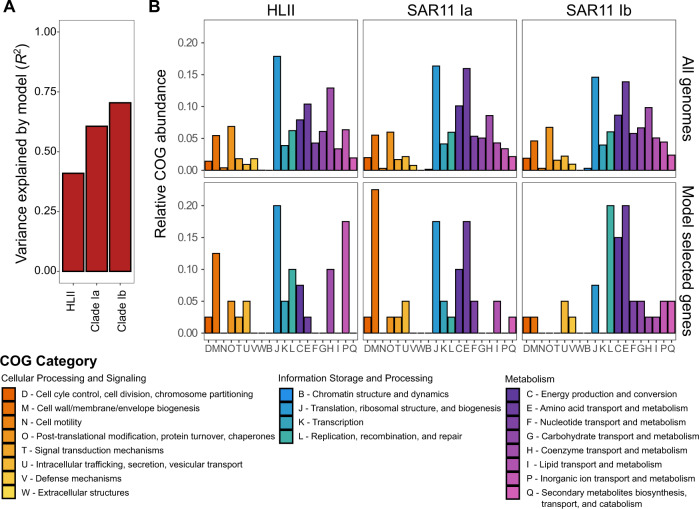
Coverage of a sparse set of explanatory genes related to cell structure, replication, energy production, amino acid, and inorganic ion transport predicts replication patterns of *Prochlorococcus* and SAR11 ecotypes in random-forest models. **A** The variance explained (*R*^2^) by the random-forest models for the HLII, clade Ia, and clade Ib random-forest models. **B** Comparison of the relative frequency of genes annotated by their Cluster of Orthologous Groups (COG) category for all reference genomes within a specific ecotype and for the 40 explanatory genes selected by the random-forest model.

Genomic strategies related to nutrient uptake and transport were strongly predictive of estimated replication. We employed a random-forest analysis in order to take an agnostic approach to identifying explanatory cellular processes. For each ecotype, random-forest models selected 40 genes that explained 41.0, 60.6, and 70.4% of the variance in replication patterns for *Prochlorococcus* HLII and SAR11 clade Ia and clade Ib ecotypes, respectively (Figs. 5A and S11). These genes were evenly spread across reference genomes and were largely multi-copy core genes, suggesting that genes which are shared widely throughout populations tend to be more informative of replication (Fig. S12). The presence of genes linked to inorganic ion transport and metabolism (COG category P) separated populations with different *R*_24hr,max._. For example, HLII replication was explained by and negatively correlated with the coverage of genes to the transport of key trace metals (Zn/Mo) and phosphate/phosphonate (Fig. S11). Through its structural and catalytic role, Zn is known to be associated with DNA replication and organic P assimilation, and Mo is associated with photorepair and molydopterin biosynthesis that is important for nitrate assimilation [57–59]. Similar to the GAM analysis, negative correlations between *R*_24hr,max._ and P uptake genes suggest that low P availability may limit HLII replication. This result is consistent with previous metagenomic analyses, which identified phosphate-binding proteins and phosphate ABC-transporters as having some of the largest biogeographic differences in coverage for both *Prochlorococcus* and SAR11 [60]. In contrast to HLII, SAR11 Ia and Ib random-forest models had an enrichment of genes related to amino acid transport and metabolism (COG category E) (Fig. 5). In SAR11 Ia, a number of ABC-type amino acid transport genes explained *R*_Obs_. In addition, the gene threonine dehydratase, an enzyme responsible for catalyzing the conversion of L-threonine into alpha-ketobutyrate and ammonia, was identified as the most explanatory gene (high MDA) (Fig. S11). Similarly, aminotransferase was identified as an important explanatory gene for SAR11 Ib. In combination with the GAM analysis, which suggests that SAR11 is adapted to regions with low inorganic N supply, this data suggests that N-stress in SAR11 may be partially overcome by organic N assimilation. Preliminary research suggests that organic sources of N such as amines can meet the N requirement of SAR11 strains [61]. Overall, these results independently identify nutrient genes as being highly predictive of *Prochlorococcus* and SAR11 ecotype replication.

Genes related to growth, energy production, and genomic replication were also linked to shifts in *R*_Obs_ and *R*_24hr,max._, suggesting that this metric is related to key genomic adaptations and cellular processes. For example, SAR11 Ib *R*_Obs_ was predicted by genes related to cellular respiration including lipid metabolism, dehydrogenases, ATP synthase, and cytochrome c biogenesis (COG category C) (Fig. 5). HLII and SAR11 Ia random-forest models were enriched in genes related to cell wall, membrane, and envelop biogenesis (COG category M). Finally, HLII and SAR11 Ib replication was explained by genes in the replication, recombination, and repair COG category (L) including DNA repair ATPase gene RecN and ribonuclease HII. The link between *R*_24hr,max._ or *R*_Obs_ and fine-scale genomic content related to energy production and conversion as well as cell wall structure further anchors replication trends in cell physiology, and suggests that this metric may be informative of cellular processes that influence ecosystem-level biogeochemical cycles.

An important consideration for our metagenomic replication estimate is that it reflects gross, rather than net, population growth. Without a loss term due to cell death or sinking export, genomically estimated replication may have a variable relationship with net population-level changes [33]. However, we found that two factors strongly influenced our replication predictions, which, to our knowledge, have not been accounted for in previous bioinformatic pipelines or analyses. First, sequencing depth was highly correlated with coverage slope (*p* value < 0.0001) (Fig. S2). This may be due, in part, to an increased number of reads leading to a greater proportion mapped to the origin of replication. Second, replication estimates were strongly influenced by synchronized cell cycle regulation (Fig. 1). This result is consistent with a recent laboratory-based study of *Synechococcus* cultures [34]. *Synechococcus* demonstrates a much more diverse diel cell cycle compared to *Prochlorococcus* [62], nevertheless, the work by Carroll et al. clearly emphasizes the necessity of accurately identifying the peak in replication for light-synchronized populations in order to link the maximum percent of cells in S-phase with growth rates. Thus, applying a Bayesian model to determine both the daily peak in replication and associated error rates was critical for our assessment of the biogeography of genomically estimated *Prochlorococcus* replication. However, as a result, samples collected during large parts of the day were uninformative for this clade and the spatial resolution of our HLII *R*_24hr,max._ is much lower than the resolution of SAR11 *R*_Obs_ (i.e., daily versus hourly). Finally, the analysis presented here is corroborated by a significant correlation between estimated *Prochlorococcus* HLII replication and autotrophic C uptake (Fig. S7). The observed relationship is notable given that whole community autotrophic C uptake measurements (and concurrent flow cytometry measurements) were initiated between 07:00–13:25 [37] and thus did not always overlap with the time of maximum *Prochlorococcus* replication. Significant advances have been made in high-resolution daily estimates of marine cyanobacteria growth rates using flow cytometry [16, 36]. However, due to the flow cytometry sampling design on I09N and the lack of flow cytometry data on I07N and C13.5, neither *Prochlorococcus* growth rates from diel forward scatter data nor time-appropriate cell cycle calculations of the maximum percent of cells in S-phase can be performed using this dataset. Thus, further ground truthing of this metric is required and future questions regarding the relationship between ecotype-specific replication and genus-level growth will significantly benefit from concurrent measurements of a variety of growth rate estimates in both in situ and laboratory studies.

## Conclusions

We find that by anchoring our metagenomic estimate of ecotype-specific replication in genome structure and accounting for sequencing biases, we detected replication patterns reflecting in situ biological processes. Both *Prochlorococcus* HLII and HLI showed significant diel rhythmicity with peak *R*_Obs_ occurring between 19:00–22:00 (Fig. 1), consistent with previously characterized cell cycles [35, 53, 63]. Although SAR11 did not demonstrate diel fluctuations in replication, the ecotypes we examined exhibited consistent bi-linear coverage patterns throughout the day across ocean basins (Fig. S4). Additionally, systematic and quantitative spatial patterns in estimated replication were strongly linked to temperature, nutricline depth, and nutrient stress (Figs. 3–5). Both diel and spatial replication trends were robust across multiple ocean basins with few outliers, suggesting that stochasticity in coverage slopes driven by processes such as ultraradian growth or multiple replication forks in slow-growing taxa had limited effect in the observed populations [64]. Thus, a distinct advantage of our methodology is the ability to resolve the instantaneous, in situ relative replication patterns of individual taxa. However, our replication estimate is dependent on the availability of a reference genome sharing high synteny of single-copy core genes with in situ populations. In the absence of such reference genomes, other metrics of metagenomically estimated, population-resolved in situ microbial activity can be extrapolated from genomic signatures (e.g., codon usage bias), but cannot provide a time-resolved estimate of in situ replication [65, 66]. Given the various limitations across methodologies, users of metagenomic replication estimates should carefully consider appropriate sampling strategies with bioinformatic normalization techniques. Nevertheless, the method described here may be particularly applicable to bacteria that demonstrate potential ecotype phylogenetic structure and high microdiversity in other systems, such as *Curtobacterium* in soil systems [67] and *Klebsiella* and *Enterococcus faecalis* in human gut microbiomes [68]. Overall, with temporally appropriate, high-resolution sampling, and deep sequencing efforts, we can calculate relative taxa-specific replication to relate to cellular, ecological, and biogeochemical processes.

The observed temporal and spatial biogeography of microbial replication occurred at fine scales of diversity and provided an added dimension to our understanding of both the niche space and genomic adaptations of *Prochlorococcus* and SAR11 ecotypes. Replication patterns showed systematic changes on a global scale (Fig. 3) that differed from metagenomic read frequencies (Fig. 2). Variability in estimated replication (Fig. 3) is consistent with modeled estimates of ecotype growth [54] and supported by community-level measurements of productivity. Specifically, HLII replication showed a significant relationship with autotrophic C uptake that was regionally dependent (Fig. S7). Although this relationship was limited to a small number of observations, it emphasizes that the relative contribution of HLII to primary production may be spatially variable and dependent on the environmental regulation of both cell abundance and growth rate [37, 69]. In addition, our replication metric revealed novel insights into the adaptive traits delineating the biogeographic distribution of microbial niche space. For instance, the HLII ecotype is adapted to high-light, high-temperature environments [8, 10]. However, our results indicated that nutrient availability played a stronger role in regulating HLII replication (Figs. 4 and 5). In contrast to *Prochlorococcus*, a combination of high microdiversity and potential mixotrophy has previously made classifying ecotype-level niche partitioning and adaptation in the SAR11 clade more complex [70]. Nevertheless, SAR11 ecotypes demonstrated habitat-specific partitioning in replication strategies for surface ocean communities. Specifically, clade Ia had the highest estimated replication in regions with deep nutriclines (~100 m) and intermediate temperatures (25–27 °C), whereas clade Ib had highest estimated replication in regions with shallower nutriclines (~50 m) and high temperatures (28–30 °C) (Fig. 4). Finally, these patterns were strongly linked to gene content related to cell wall structure, energy production, and nutrient uptake (Fig. 5). In sum, the results presented here show that metagenomically estimated, ecotype-level replication patterns are strongly influenced by the interaction between genomic adaptation and environmental gradients across ocean basins. In the future, this technique may help further elucidate genotype-resolved microbial responses to environmental change across systems.

## Supplementary information

Supplementary Information

## Data Availability

Raw metagenomic reads are available via the National Center for Biotechnology Information Sequence Read Archive (BioProject ID PRJNA656268). Metadata are available via https://cchdo.ucsd.edu. A complete description of Bio-GO-SHIP metagenomes and associated metadata is available via [52]. Supplementary information is available at The ISME Journal’s website. Analysis code is available via https://github.com/aalarkin/SCCG-Replication-Slope and https://github.com/georgehagstrom/MetagenomeGrowthModel.
