# Supplementary material for: Basin-scale biogeography of Prochlorococcus and SAR11 ecotype replication
Source: ISME J. 2022 Oct 22;17(2):185–94. doi: 10.1038/s41396-022-01332-6 (PMC9589681; doi:10.1038/s41396-022-01332-6)
Supplement: Supplementary file 1 — Supplementary Information [file 41396_2022_1332_MOESM1_ESM.pdf]

Supplementary Information:
Basin-scale biogeography of *Prochlorococcus* and SAR11 ecotype replication

Alyse A. Larkin[1], George I. Hagstrom[2], Melissa L. Brock[3], Nathan S. Garcia[1], and Adam C. Martiny[1,3*]

[1]Department of Earth System Science, University of California, Irvine, CA
[2]Department of Ecology and Evolutionary Biology, Princeton University, NJ
[3]Department of Ecology and Evolutionary Biology, University of California, Irvine, CA

*Corresponding Author: Adam C. Martiny; 3200 Croul Hall, University of California at Irvine, Irvine, CA, 92697, USA; 949-824-9713; amartiny@uci.edu

Supplemental Methods

DNA Extraction and Library Preparation

DNA extraction and metagenomic library prep are fully described in (1). Briefly, DNA was extracted via lysozyme (4.35 mg mL^{-1} final conc.), Proteinase K (0.1 mg mL^{-1} final conc.) and SDS buffer (0.5% final conc.) incubation. Next, DNA was precipitated with sodium acetate (245 mg mL−1, pH 5.2) and ice-cold isopropanol (100%), and pelleted via centrifuge at 15,000 × g at 4°C for 30 mins. Finally, DNA was resuspended in TE buffer (10 mmol L^{-1} Tris-HCl, 1 mmol L^{-1} EDTA), purified using a Zymo genomic DNA Clean and Concentrator kit (Zymo Research Corp., Irvine, CA), and diluted to 2 ng/µl in Tris-HCl buffer (10 mmol L^{-1}, pH 8.0).

A total of 2 ng DNA from each sample was added to 1.5 µl tagmentation reactions and incubated at 55°C for 10 min. Unique dual index (UDI) barcodes (8 bp) were annealed via polymerase chain reaction (2.04 µM UDI barcodes [forward and reverse], 204 µM dNTPs, 0.0204 U Phusion High Fidelity DNA polymerase and 1.02X Phusion HF Buffer [ThermoFisher, Waltham, MA]) using the following protocol: 72°C for 2 min., 98°C for 30 s., followed by 13 cycles of 98°C 10 s., 63°C 30 s., 72°C 30 s., and 72°C for 5 min. Libraries were size-selected using a buffered solution (58.4 mg mL^{-1} NaCl, 1 mmol L^{-1} EDTA, 10 mmol L^{-1} Tris-HCl, 180 mg mL^{-1} PEG-8000, 0.055% Tween-20 final conc.) of Sera-mag SpeedBeads (ThermoFisher, Waltham, MA) at a 1:1 product-to-bead volume ratio. Libraries were quantified using a Qubit dsDNA HS Assay kit (ThermoFisher, Waltham, MA) and a Synergy 2 Microplate Reader (BioTek, Winooski, VT, USA) and pooled at equimolar concentrations. Library pools were then analyzed via KAPA qPCR (Roche, Basel, Switzerland) and a 2100 Bioanalyzer high sensitivity DNA trace (Agilent, Santa Clara, CA) prior to sequencing. I09N, I07N, and C13.5 samples were pooled separately and sequenced on Illumina NovaSeq lanes using 150 bp paired-end chemistry with 300 cycles (Illumina, San Diego, CA).

Metagenome QC and Mapping Parameters

Illumina adapters were removed and sequences were quality filtered using Trimmomatic (v0.35). Parameters: PE ILLUMINACLIP:NexteraPE-PE.fa:2:30:10 SLIDINGWINDOW:4:15 MINLEN:36

BBMap (v37.50) was used to remove any potential PhiX contamination.
Parameters: bbduk.sh -Xmx1g ref=/resources/phix174_ill.ref.fa.gz k=31 hdist=1

Bowtie2 (v2.2.7) was used to map metagenomic reads to reference genomes.
Parameters: --local -D 15 -R 2 -L 15 -N 1 –gbar 1 –mp 3

Anvi'o (v5.5) was used to create a pangenome database of all reference genomes.
Parameters: anvi-pan-genome --minbit 0.5 --mcl-inflation 10 --use-ncbi-blast

Daily HLII Maximum Replication

To estimate daily maximum replication ($R_{24hr,max.}$) of the HLII ecotype, we developed a hierarchical/multi-level model which predicts $R_{24hr,max}$ using R_{Obs} and the measurement times, fitting unknown parameters using Bayesian statistics (2). The model assumes the temporal variation of the expectation of *M* over the diel cycle follows a Gaussian function:

$$RepSlope(t, t_1, day) = c_0 + (R_{24hr,max}(day) - c_0)\, exp((t - t_1)^2/(2t_w^2))$$

Here c_0 is the baseline replication slope observed outside the typical replication window, t is the time of day in units of hours and starting at solar noon, t_1 is the time of maximum replication slope, t_w is the width of the time interval over which replication occurs, and $R_{24hr,max}(day)$ is the maximum of the replication slope occurring within the noon-to-noon bin represented by the value of the variable day. The RepSlope function is diagramed in Figure S4C. Then we use the following statistical model for R_{Obs} and $R_{24hr,max}$:

$$R_{24hr,max}(day) \sim \mathrm{NormalT}(R_{24hr,max0}, 0.3, lower = 0.0)$$
$$R_{24hr,max0} \sim NormalT(0.2, 1.0, lower = 0.0)$$
$$R_{obs} \sim Normal(RepSlope(R_{24hr,max}(day), t_{obs}, t_1, t_w), sigma\ \ )$$
$$t_1 \sim NormalT(10.0, 2.0, lower = 0.0, upper = 24.0)$$
$$sigma \sim Uniform(lower = 0.0, upper = 1.0)$$
$$t_w \sim Uniform(lower = 1.0, upper = 6.0)$$
$$c_0 \sim NormalT(0.2, 1.0, lower = 0.0)$$

This models the value of each R_{Obs} as a normal distribution with mean given RepSlope evaluated at the observation time, with a value of $R_{24hr,max}$ distributed normally across each noon-to-noon bin, and with weakly informative priors on the replication time t_1, the width of the replication period t_w, the background replication slope c_0, and the mean maximum replication slope $R_{24hr,max}$. The posterior distribution of model parameters was computed using Markov-Chain Monte Carlo (MCMC) methods, in particular the Hamiltonian MC algorithm based on the No-U-Turn Sampler (NUTS) (3, 4) implemented in the Stan probabilistic programming language (5). 24 Markov Chains were generated, each consisting of 2000 iterations, the first 1000 of which were discarded as part of the burn-in period, leading to 24000 independent samples from the posterior distribution. To check for convergence of the Markov Chains and efficient exploration of the posterior distribution, several diagnostics tests were performed, including verifying the value of $\hat{R}$ statistic was less than 1.01 and that the effective sample size was close to the number of samples (6), that the estimated Bayesian fraction of missing information was greater than 0.2 for all chains, and that no divergent transitions occurred during the sampling procedure (7). Pair-plots of the posterior distribution for model parameters are shown in Figure S4A, and a table showing summary statistics from the inference procedure and the values of $\hat{R}$ and the effective sample size are given in in Figure S4B.

Supplemental References

1. Larkin AA, Garcia CA, Garcia N, Brock ML, Lee JA, Ustick LJ, et al. High spatial resolution global ocean metagenomes from Bio-GO-SHIP repeat hydrography transects. Scientific Data. 2021;8(1).
2. Gelman A, Carlin JB, Stern HS, Dunson D, Vehtari A, Rubin DB. Bayesian data analysis: Chapman and Hall/CRC; 2013.
3. Neal RM. MCMC Using Hamiltonian Dynamics. Handbook of Markov Chain Monte Carlo. 2011:113-62.
4. Hoffman MD, Gelman A. The No-U-Turn Sampler: Adaptively Setting Path Lengths in Hamiltonian Monte Carlo. Journal of Machine Learning Research. 2014;15:1593-623.

5. Team SD. Stan Modeling Language User's Guide and Reference Manual. 2.28 ed2021.
6. Vehtari A, Gelman A, Simpson D, Carpenter B, Bürkner P-C. Rank-normalization, folding, and localization: An improved R-hat for assessing convergence of MCMC. arXiv preprint arXiv:190308008. 2019.
7. Betancourt M. A conceptual introduction to Hamiltonian Monte Carlo. arXiv preprint arXiv:170102434. 2017.

Supplemental Figures

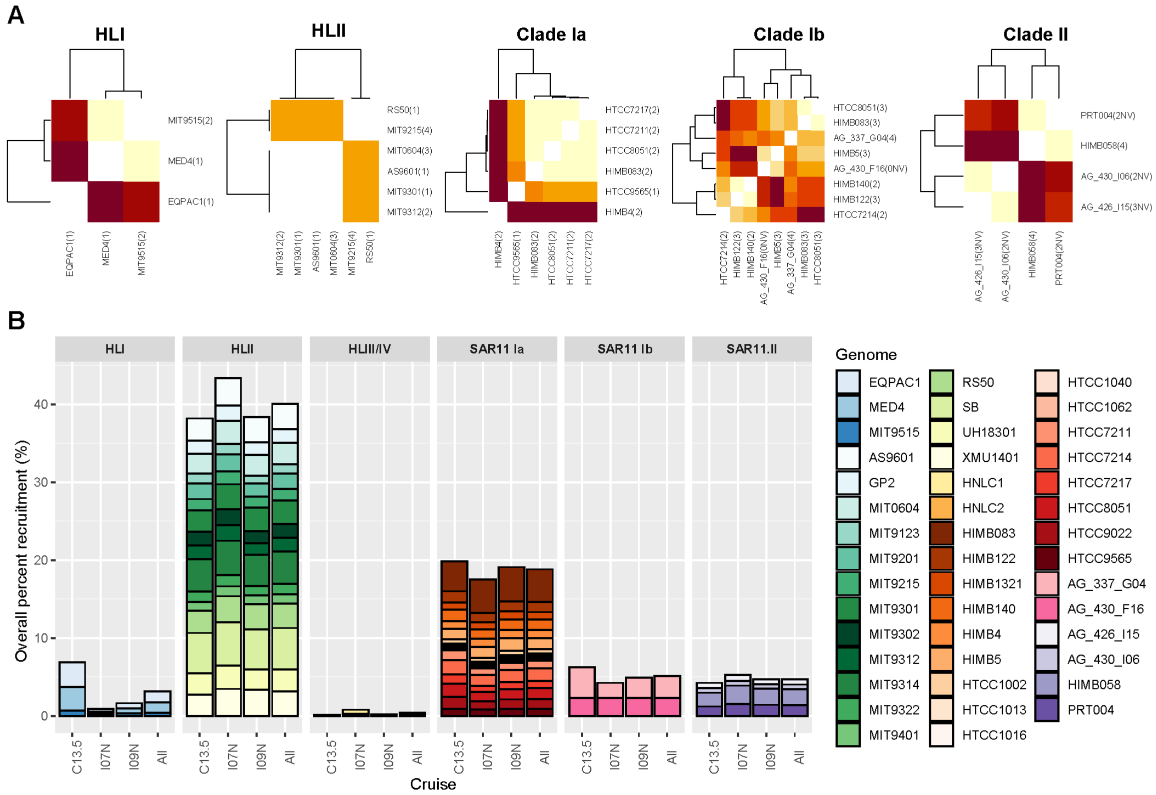

Fig. S1. Genomic synteny of single copy core genes (SCCG) and percent read recruitment were used to identify reference genomes with highly conserved SCCG order that were representative of both broader ecotypes and *in situ* communities. (A) The double cut and join (DCJ) distance of genome-ordered SCCGs was used to identify the top six genomes with the lowest mean distances within each ecotype. Only two reference genomes were available for the HLIII/IV ecotype and thus synteny-based clustering was impossible for this clade. For SAR11 clade Ib, only two single cell genomes with >90% completion were available as references at the time of the analysis, thus the clade Ia genomes with the highest read recruitment were used as potential reference genomes for this clade. The DCJ distance for the top 6 genomes was clustered using average linkage hierarchical clustering. The value in the parentheses represents the number of gaps identified in mapped SCCGs. In addition, “NV” indicates that no v-shaped coverage pattern could be identified. (B) The overall percent recruitment of all reads mapped to each genome across each cruise or all combined cruises.

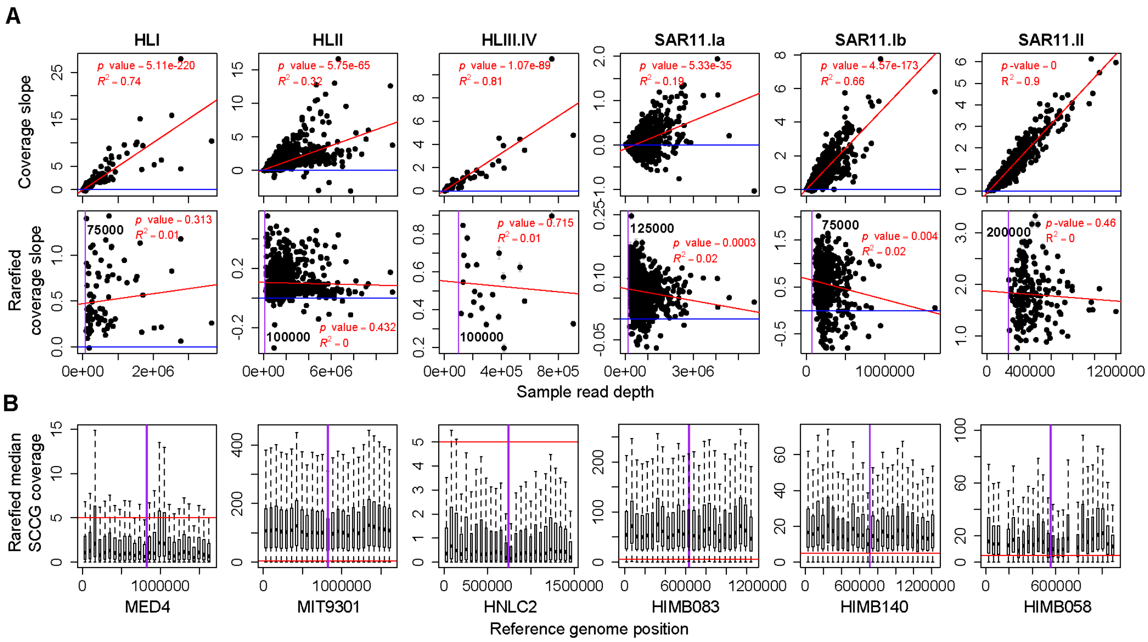

Fig. S2. (A) Comparison of the replication slope and the total reads mapped to the top six most abundant *Prochlorococcus* and SAR11 ecotypes across the C13.5, I07N, and I09N transects for the full (top row) and rarefied (bottom row) datasets. Red lines and text represent linear regressions between reads mapped and replication slope with corresponding R^2 and *p*-values. Purple vertical lines represent the selected rarefaction depth (labeled in bold). Blue lines delineate positive versus negative slopes. (B) Summed single copy core gene (SCCG) coverage mapped to reference genome position for all stations. Red line represents 5X coverage cut off. Vertical purple line represents the estimated terminus of replication.

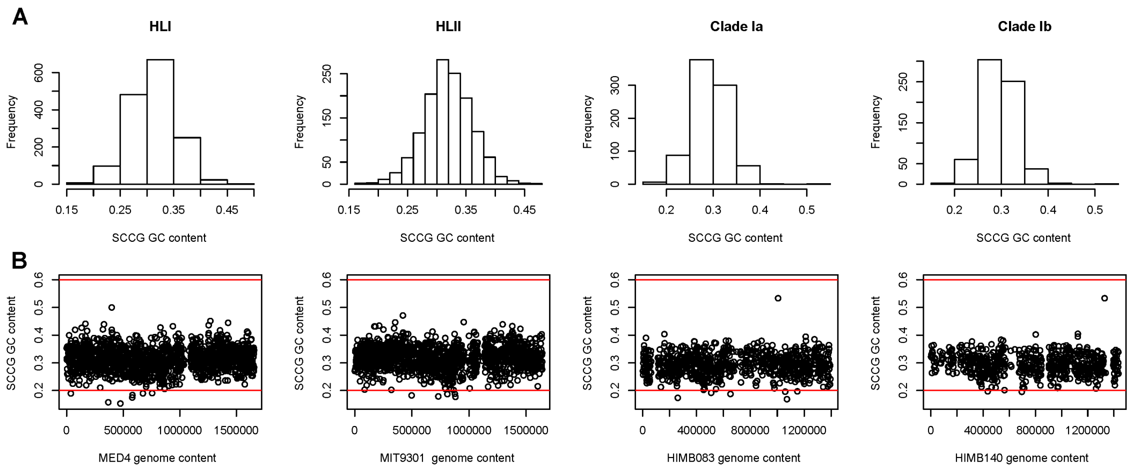

Fig. S3. (A) GC content frequency for single copy core genes (SCCG) from *Prochlorococcus* HLI and HLII ecotypes and SAR11 clade Ia and clade Ib ecotypes. (B) SCCG GC content as mapped to reference genome position for HLI, HLII, clade Ia, and clade Ib. Red lines represent GC content of 0.2 and 0.6, below and above which, respectively, the Illumina NovaSeq platform demonstrates sequencing bias.

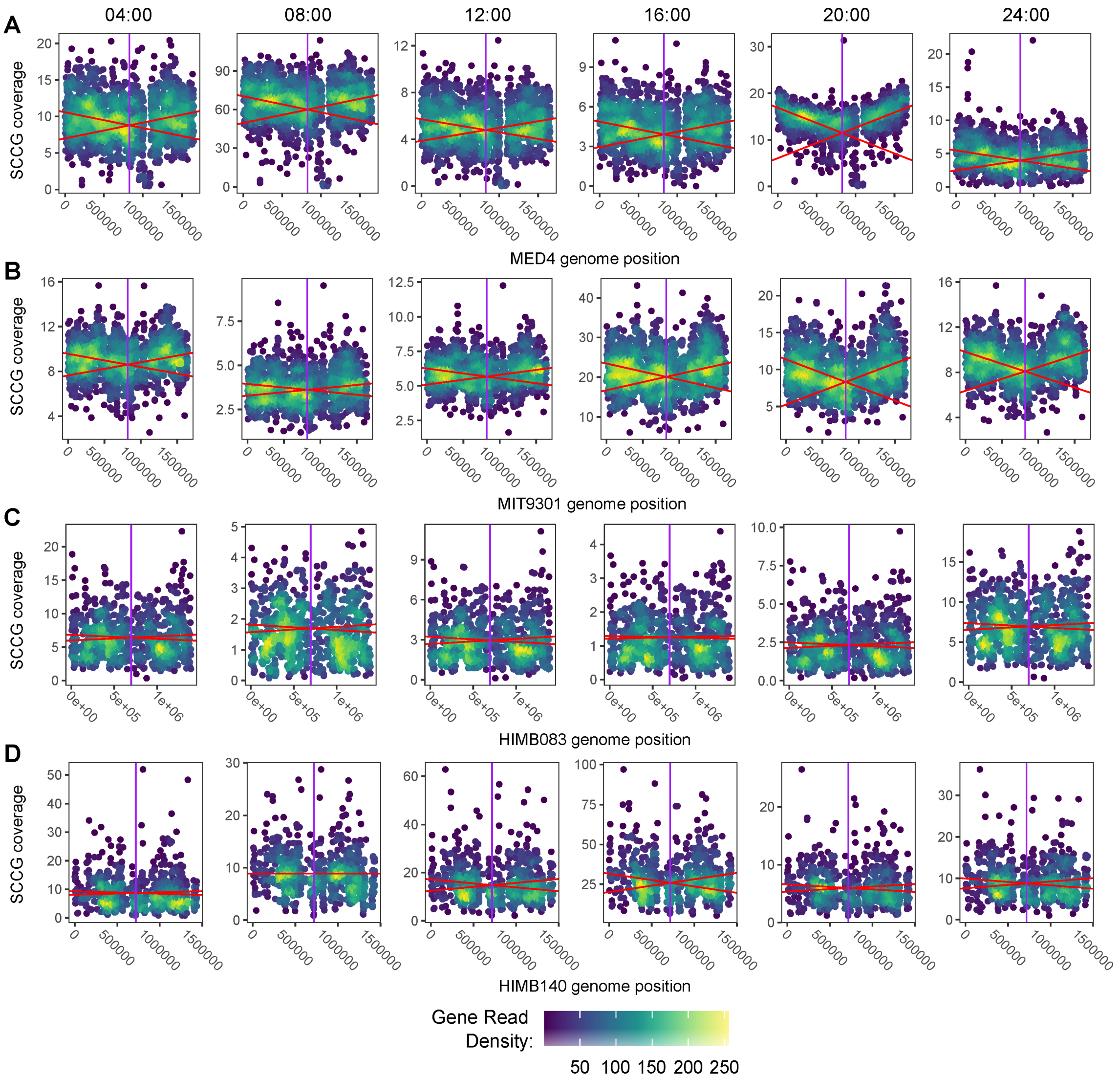

Fig. S4. Single copy core gene (SCCG) read coverage mapped to reference genome position for the ecotypes (A) *Prochlorococcus* HLI, (B) *Prochlorococcus* HLII, (C) SAR11 clade Ia, and (D) SAR11 clade Ib across the 24-hour day cycle. At each time point across the diel cycle (04:00, 08:00, 12:00, 16:00, 20:00, 24:00), the sample with the smallest residual to the smoothed central tendency of R_{Obs} across all samples (see Figure 1, main text) was selected as representative. The purple line is the terminus of replication. The red line is the bi-linear fit to the coverage pattern.

(A) Posterior Pair Plot

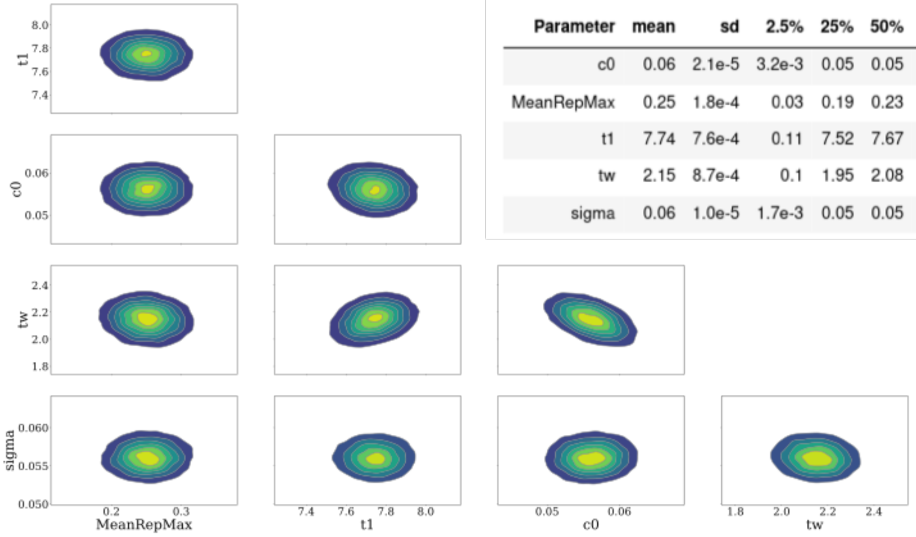

(B) Model Parameters

Parameter	mean	sd	2.5%	25%	50%	75%	97.5%	n_eff	Rhat
c0	0.06	2.1e-5	3.2e-3	0.05	0.05	0.06	0.06	0.06	23738
MeanRepMax	0.25	1.8e-4	0.03	0.19	0.23	0.25	0.27	0.32	33370
t1	7.74	7.6e-4	0.11	7.52	7.67	7.74	7.81	7.95	20746
tw	2.15	8.7e-4	0.1	1.95	2.08	2.15	2.21	2.34	13216
sigma	0.06	1.0e-5	1.7e-3	0.05	0.05	0.06	0.06	0.06	27900

(C)

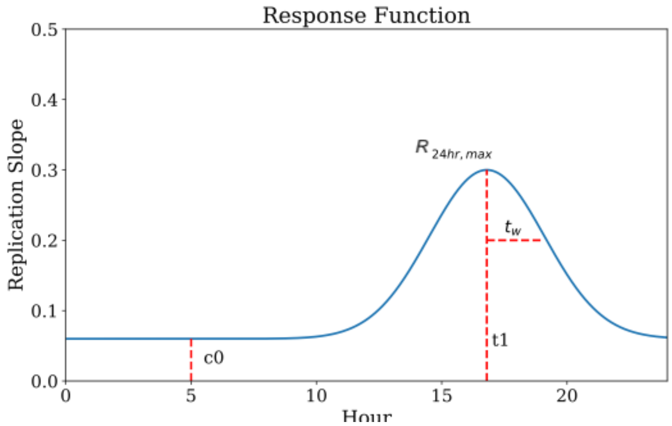

Fig. S5. (A) Pair-plot of the posterior distribution of key model variables. (B) Table giving summary statistics generated from the Markov Chains and values of key diagnostics. (C) Illustration of the RepSlope function which models how R_{obs} depends on time.

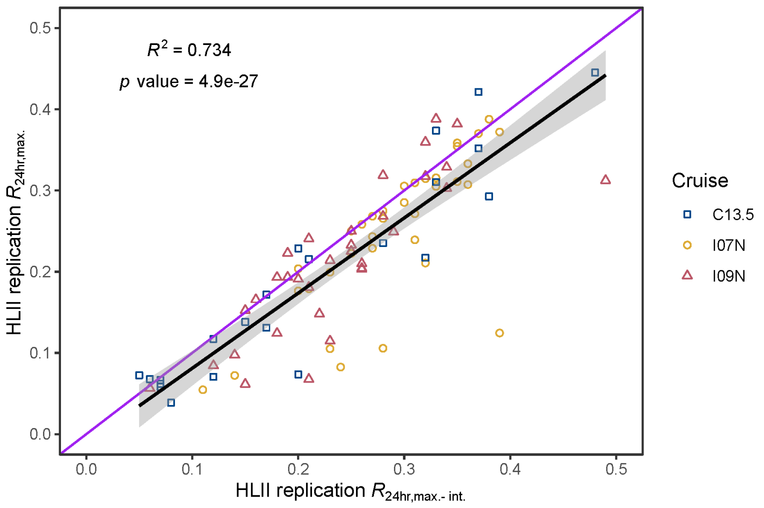

Fig. S6. Daily replication maximum ($R_{24hr,max.}$) for *Prochlorococcus* HLII as predicted by the RepSlope function model as compared to daily replication maximums as estimated via linear interpolation ($R_{24hr,max – int.}$) from the nearest observations (i.e., $R_{Obs.}$) across the C13.5, I07N, and I09N cruise transects.

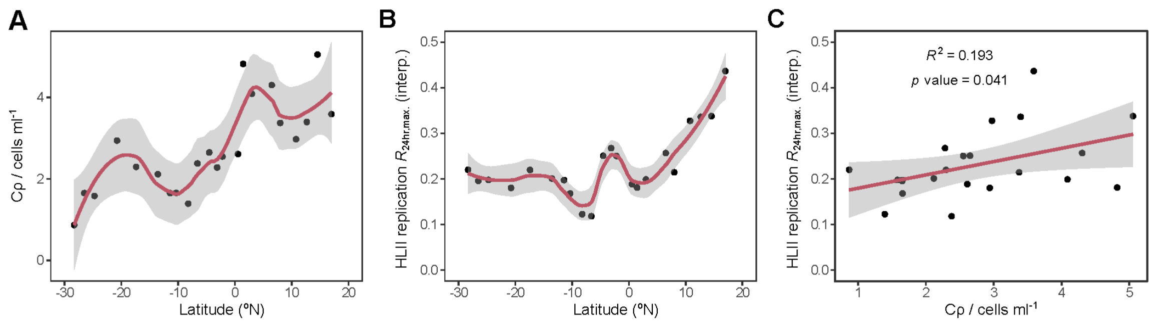

Fig. S7. Normalized C uptake (Cρ, nmol C L^{-1} percent daylight^{-1}) per *Prochlorococcus* cell (cells ml^{-1}) (i.e., fmol C percent daylight^{-1} cell^{-1}) and the HLII $R_{24hr,max.}$ as interpolated to the corresponding C uptake incubation location across the I09N transect. Red lines in (A) and (B) represent the smoothed Loess curve trend (span = 0.25) across the I09N latitudinal transect. The red line in (C) represents the linear regression between C uptake and HLII replication. Grey shading represents 95% confidence intervals.

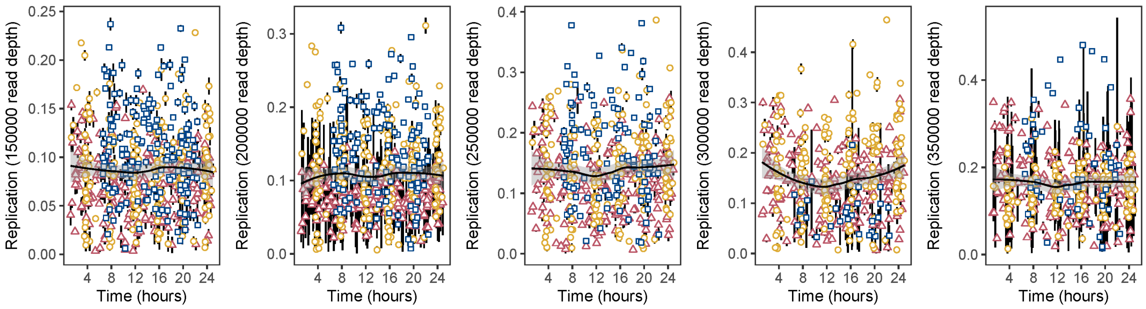

Fig. S8. Diel replication (R_{Obs}) trends for SAR11 clade Ia across I07N (yellow circles), I09N (red triangles), and C13.5 (blue squares) rarefied to five different sequence depths (150000, 200000, 250000, 300000, and 350000 reads per sample, i.e. ~5x – 15x coverage). Point whiskers represent the standard error of replication estimates across 3x rarefactions.

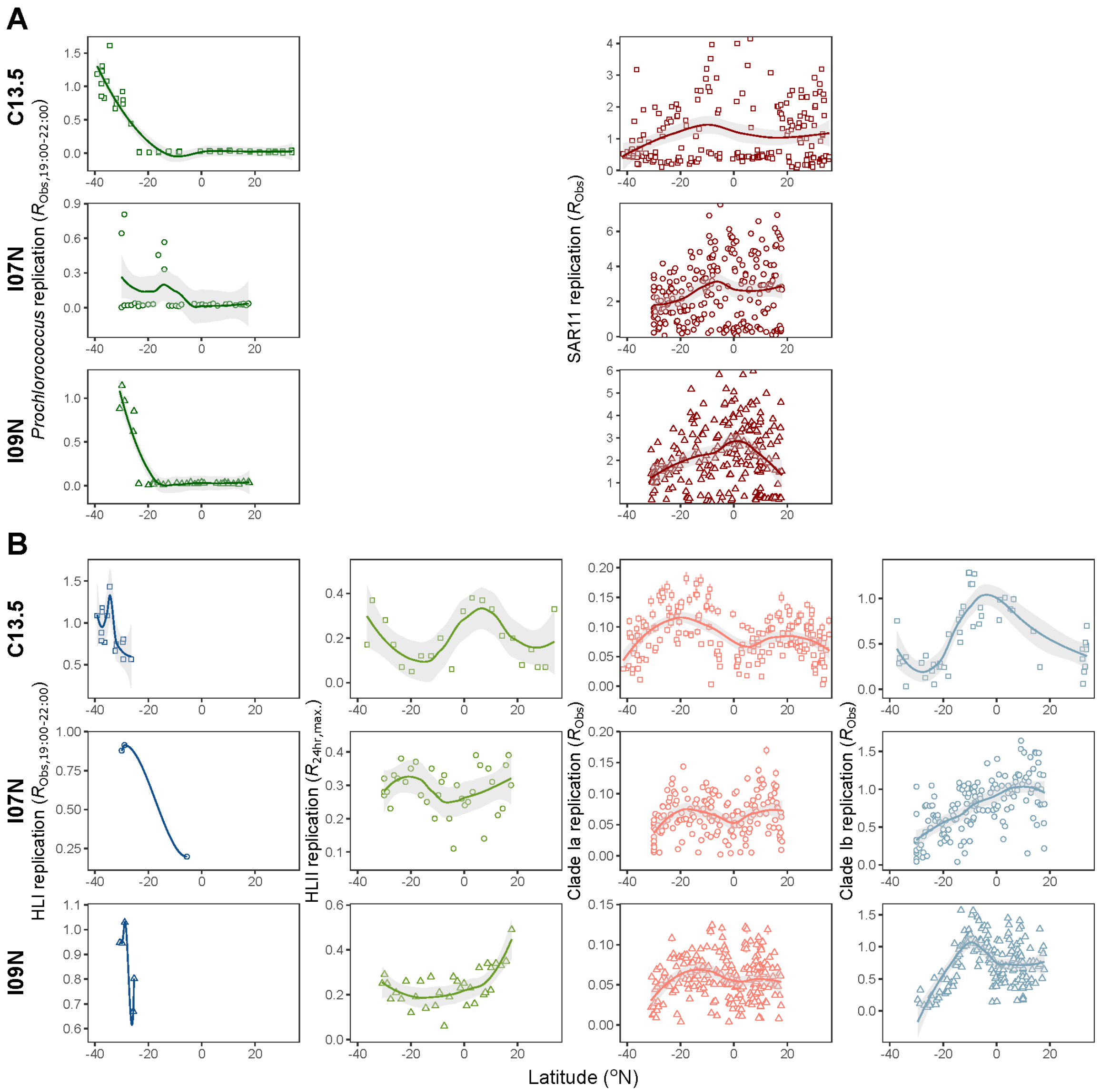

Fig. S9. Biogeography of (A) genus level *Prochlorococcus* ($R_{Obs,19:00-22:00}$) and SAR11 (R_{Obs}) replication as well as (B) ecotype level HLI ($R_{Obs,19:00-22:00}$), HLII ($R_{24hr,max.}$), clade Ia (R_{Obs}), and clade Ib (R_{Obs}) replication across the Bio-GO-SHIP C13.5, I07N, and I09N ocean sections. Samples with less that 5X genome coverage or negative slopes were excluded from the analysis.

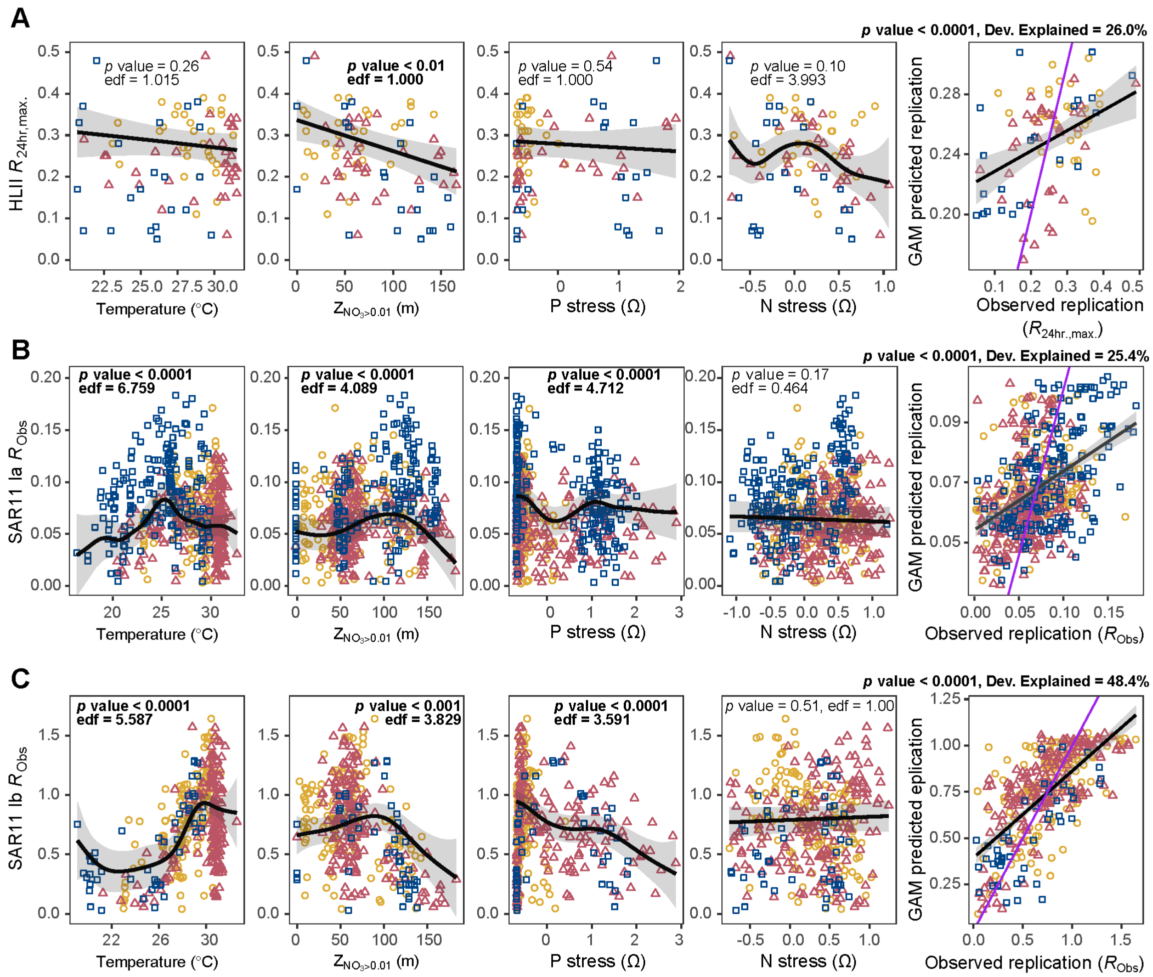

Fig. S10. General additive models (GAMs) reveal bottom-up controls of temperature and nutrient supply on replication across ocean basins. GAM-identified relationships between temperature, nutricline depth, P-stress, N-stress, and (A) HLII ($R_{24hr,max}$), (B) SAR11 clade Ia (R_{Obs}), and (C) SAR11 clade Ib (R_{Obs}) are depicted for C13.5 (blue squares), I07N (yellow circles), and I09N (red triangles) combined. Black lines represent predicted GAM smooths with 95% confidence intervals in grey. The significance of individual smooths is depicted within the first four columns of plots and the overall GAM significance and deviance explained for each ecotype is depicted over each row. Significant relationships are bolded. Scatter plots in the far-right column depict the relationship between GAM-based predictions and observed *R* values. Black lines represent linear regressions with 95% confidence intervals in grey. Purple lines represent the 1:1 line.

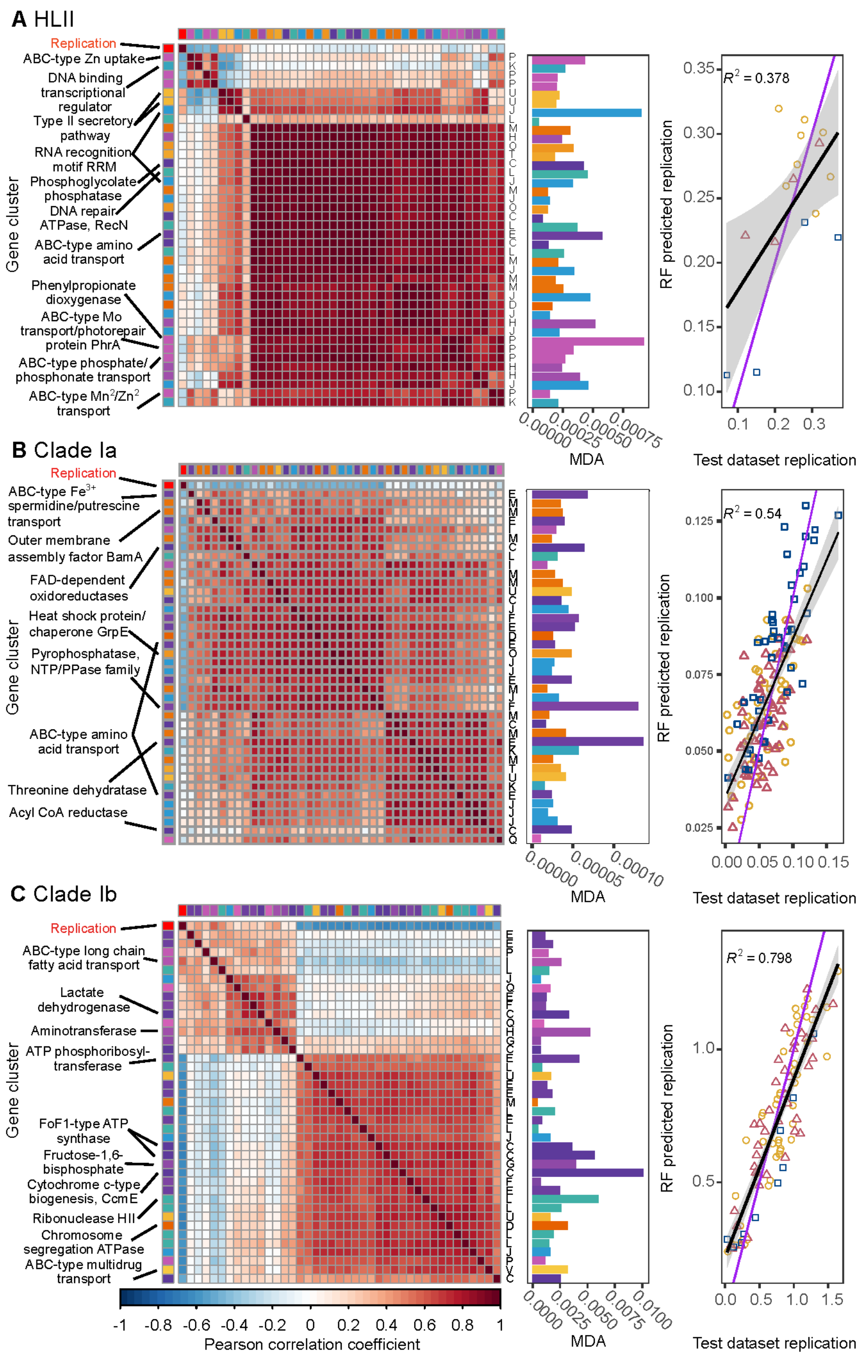

Cellular Processes and Signaling
- D - Cell cyle control, cell division, chromosome partitioning
- M - Cell wall/membrane/envelope biogenesis
- N - Cell motility
- O - Post-translational modification, protein turnover, chaperones
- T - Signal transduction mechanisms
- U - Intracellular trafficking, secretion, vesicular transport
- V - Defense mechanisms
- W - Extracellular structures
- Y - Nuclear structure
- Z - Cytoskeleton

Metabolism
- C - Energy production and conversion
- E - Amino acid transport and metabolism
- F - Nucleotide transport and metabolism
- G - Carbohydrate transport and metabolism
- H - Coenzyme transport and metabolism
- I - Lipid transport and metabolism
- P - Inorganic ion transport and metabolism
- Q - Secondary metabolites biosynthesis, transport, and catabolism

Information Storage and Processing
- A - RNA processing and modification
- B - Chromatin structure and dynamics
- J - Translation, ribosomal structure, and biogenesis
- K - Transcription
- L - Replication, recombination, and repair

Unknown
- R - General function prediction only
- S - Function unknown

Fig. S11. Coverage of a sparse set of explanatory genes predicts replication patterns of *Prochlorococcus* and SAR11 ecotypes (A) HLII, (B) clade Ia, and (C) clade Ib in test datasets. Heatmaps depict the Pearson's correlation coefficient between replication slope and the coverage of the predictive genes determined by the random forest analysis. The genes depicted in the barplots and heatmaps are colored by their respective Cluster of Orthologous Groups (COG) categories. Barplots show the ranked feature importance of the genes used to train the optimized random forest model as mean decrease in model accuracy (MDA) if the feature is removed from the model. Scatterplots show the relationship between observed and predicted replication for a randomly selected test dataset for C13.5 (blue squares), I07N (yellow circles), and I09N (red triangles) samples. Black lines represent linear regressions with 95% confidence intervals. Purple lines represent the 1:1 line.

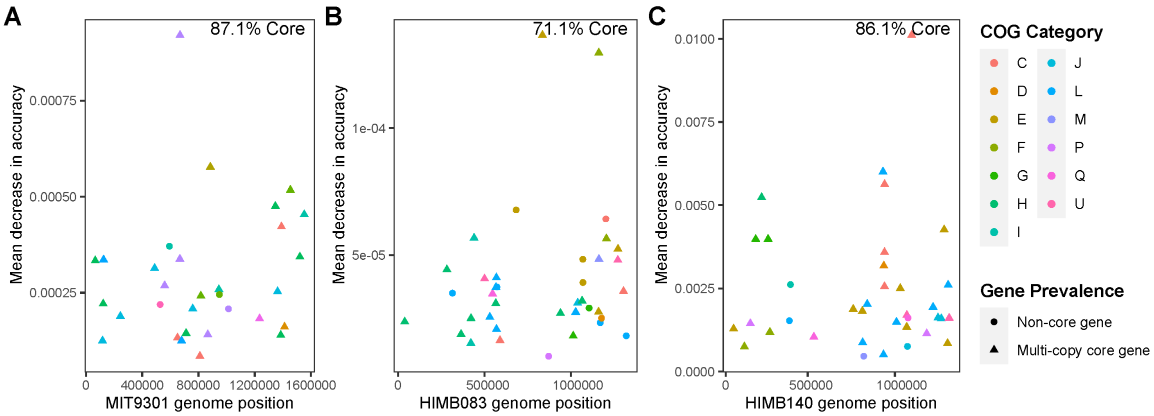

Fig. S12. Feature importance of the genes used to train the optimized random forest model as mean decrease in model accuracy (MDA) mapped to reference genome location for the ecotypes (A) *Prochlorococcus* HLII, (B) SAR11 clade Ia, and (C) SAR11 clade Ib. Colors represent the COG category of the gene and shapes represent whether or not the gene is a core gene found in all ecotype-specific reference genomes (i.e. the gene prevalence). The percentage of genes found to be core genes is denoted at the top of each plot.
